# Awareness of Chorioamnionitis and Its Complications: A Cross-Sectional Study Among Women in Makkah City

**DOI:** 10.7759/cureus.95771

**Published:** 2025-10-30

**Authors:** Yasir Katib, Mariah Almehmadi, Anwar Salem, Noor S Alharbi, Layan Alasiri, Nusaybah Shafi, Zainab Patwa, Ertwaa Alamri

**Affiliations:** 1 Medicine, Umm Al-Qura University, Makkah, SAU; 2 Medicine and Surgery, Umm Al-Qura University, Makkah, SAU; 3 College of Medicine, Umm Al-Qura University, Makkah, SAU

**Keywords:** chorioamnionitis, maternal health, pregnancy complications, premature birth, prenatal education, saudi arabia

## Abstract

Introduction

Chorioamnionitis (CA) is a pregnancy complication that can harm both mothers and newborns. This study aimed to assess the awareness and knowledge of CA among women in Makkah, Saudi Arabia.

Methodology

A cross-sectional study was conducted using an online questionnaire distributed via social media. The study included 702 women who were either currently pregnant or had been pregnant before. Participants answered questions on sociodemographic data, awareness, sources of information, and knowledge about CA. Statistical analysis was performed using IBM SPSS Statistics Version 27 (IBM Corp., Armonk, USA).

Results

Among 702 participants, 375 (53.4%) had heard of CA. Awareness was higher among non-Saudis (42, 65.6%) than Saudis (333, 52.2%). Women with postgraduate education (74, 73.3%) were more aware than those with a high school education (35, 40.2%, p<0.001). Awareness was greater among students (30, 73.2%) and employees (208, 60.1%) than housewives (137, 43.5%, p<0.001). Participants with higher income (10,000-20,000 SAR) had better awareness (172, 62.3%) than those earning <3,000 SAR (19, 32.8%, p<0.001). Women with a history of CA were significantly more aware (104, 94.5%) than those without (271, 45.8%, p<0.001). Cesarean section was more common among women with CA (93, 97.9%) than those without (2, 2.1%, p=0.003).

Conclusions

Despite moderate awareness of CA, misconceptions about its causes and treatment persist. Education, income, and previous pregnancy complications were significant factors influencing knowledge. Health campaigns and improved medical education are needed to enhance understanding and reduce pregnancy-related complications.

## Introduction

Chorioamnionitis (CA) is a common pregnancy complication involving inflammation of the placental membranes and chorion. It is also known as intra-amniotic infection, often caused by ascending bacterial infection, typically occurring with membrane rupture but sometimes without it. Clinical and histologic CA often overlap, with histologic CA being more common and identified through microscopic placental abnormalities [1].

Several studies have identified various risk factors associated with CA. These include an extended duration of membrane rupture, prolonged labor, being a first-time mother (nulliparity), and African American ethnicity. Other contributing factors include the use of internal labor monitoring, frequent vaginal examinations during labor, the presence of meconium-stained amniotic fluid, and lifestyle factors such as smoking, alcohol consumption, or drug abuse. Additionally, conditions like immune system compromise, the use of epidural anesthesia, and infections such as group B streptococcus colonization, bacterial vaginosis, sexually transmitted genital infections, and vaginal colonization with Ureaplasma have been linked to an increased risk of developing CA [2-12].

CA has been reported in 2-4% of full-term pregnancies [3] and 40-70% of preterm pregnancies [13,14]. Another retrospective cohort study conducted in 2009 at Moffitt-Long Hospital, San Francisco, reported that CA affects 1% of term and 13-60% of preterm premature rupture of membranes (PPROMs) [15].

In addition, a study conducted in Korea in 2013 identified CA as one of the risk factors for adverse neonatal outcomes [16]. This was supported by a retrospective cohort study conducted in 2017 in Washington, which reported a definite connection between CA and fetal complications, specifically respiratory distress syndrome and perinatal death [17]. Moreover, another study found that CA was associated with an increased risk of adverse maternal outcomes following cesarean section [18].

Unfortunately, only one study was conducted in Jeddah, Saudi Arabia, in 2021 to assess knowledge of CA and its complications. It showed poor knowledge about CA and its complications among 49.1% of participants [19]. Therefore, this study aims to evaluate the awareness of CA and its complications among women living in Makkah City, Saudi Arabia, and their effects on mothers and newborns.

## Materials and methods

Study design

This study employs a quantitative descriptive cross-sectional design to assess the awareness of CA and its complications among women in Makkah City, Saudi Arabia. Conducted between December 2024 and March 2025, the research uses an online questionnaire distributed via social media platforms to reach both Saudi and non-Saudi women who are currently pregnant or have experienced at least one pregnancy. The study received ethical approval from the Biomedical Research Ethics Committee at Umm Al-Qura University (Approval No. HAPO-02-K-012-2025-02-2548, dated 18/01/2025), ensuring adherence to ethical standards in data collection and participant engagement.

Sample Size

The sample size was calculated using OpenEpi version 3.0 (released in 2013, Atlanta, GA, USA), considering the following: the population size of females living in Makkah City is estimated at more than 1 million, according to the General Authority for Statistics of the Kingdom of Saudi Arabia [20]. Therefore, the minimum sample size to achieve a 95% confidence interval (CI) and a 5% margin of error was 384. To compensate for potential data loss, the total sample size was increased to 400 participants.

Data Collection

A validated questionnaire from a previous study was distributed via Google Forms through social media platforms. It was reviewed and translated into Arabic by a certified translator using a back-to-back translation technique [19]. The questionnaire consisted of four main sections. The first section collected data on participants’ sociodemographic characteristics, pregnancy history, and medical conditions, including age, nationality, educational level, family income, employment status, total number of pregnancies, number of births, number of miscarriages, history of diseases, and experience with preterm rupture of membranes (PROM). The second section assessed participants’ awareness of CA, personal experiences, sources of information about the condition, and the method of delivery. The third section examined participants’ understanding of the potential risks and complications of CA for both the mother and the newborn. The final section evaluated participants’ knowledge of CA and identified common misconceptions.

Statistical Analysis

Descriptive statistics: Descriptive statistics were used to summarize the study variables. Categorical data were presented as frequencies (N) and percentages (%), while continuous variables were expressed as mean and SD.

Inferential statistics: For inferential statistics, the normality of continuous variables was assessed using the Kolmogorov-Smirnov test, which indicated that the data were not normally distributed. Consequently, nonparametric tests were applied. The Mann-Whitney U test was used to compare differences between two-category variables, while the Kruskal-Wallis test was used for comparisons involving multiple categorical variables. Fisher’s exact test or the Fisher-Freeman-Halton exact test was applied for categorical variables with small expected counts. Spearman’s rank correlation was performed to assess the relationship between the chorioamnionitis awareness and knowledge score and other continuous variables.

Significance value: A p-value of <0.05 was considered statistically significant for all tests.

Software: Data analysis was performed using IBM SPSS Statistics version 27.0.1 (IBM Corp., Armonk, USA). This software was used for all statistical computations, including descriptive statistics, inferential tests, and correlation analyses.

Ethical consideration

The study received ethical approval from the Biomedical Research Ethics Committee at Umm Al-Qura University (Approval No. HAPO-02-K-012-2025-02-2548, dated 18/01/2025). The questionnaire began with a clear statement assuring participants that their information would remain confidential and be used solely for research purposes.

Participants were asked to either consent or decline participation, and only those who agreed were included in the study. Each participant was assigned an individual code number for data analysis to ensure anonymity and confidentiality. There were no incentives, and participation was entirely voluntary.

## Results

Sociodemographic characteristics, pregnancy history, and medical conditions of participants

The study included a total of 702 participants, with a mean age of 36.63 years (SD: 9.74). The majority were Saudi nationals (N=638, 90.9%), while non-Saudis accounted for a smaller proportion (N=64, 9.1%). In terms of education, most participants held a bachelor's degree (N=409, 58.3%), followed by postgraduate studies (N=101, 14.4%), diplomas (N=84, 12.0%), high school (N=87, 12.4%), medium education (N=16, 2.3%), and elementary education (N=5, 0.7%). Regarding family income, the highest proportion earned was 10,000-20,000 SAR (N=276, 39.3%), followed by 3,000-10,000 SAR (N=263, 37.5%), more than 20,000 SAR (N=105, 15.0%), and less than 3,000 SAR (N=58, 8.3%). In terms of employment status, employees constituted the largest group (N=346, 49.3%), followed by housewives (N=315, 44.9%) and students (N=41, 5.8%). The mean total number of pregnancies was 3.70 (SD: 2.26), with a mean of 1.85 births at ≥24 weeks (SD: 2.22) and a mean of 0.74 miscarriages at <24 weeks (SD: 1.30). The majority of participants reported having no chronic diseases (N=522, 74.4%), while urinary tract infections were the most commonly reported condition (N=86, 12.3%), followed by diabetes (N=59, 8.4%) and vaginitis or sexually transmitted infections (N=18, 2.6%). Other conditions, including high blood pressure, lupus erythematosus, weak ovum, uterine fibroids, placenta previa, polycystic ovary syndrome/obesity, hypothyroidism, glandular disorders, epilepsy, elevated liver enzymes during pregnancy, and anemia, were each reported by only one or two participants (≤0.3%). Regarding PROM, 72.9% (N=512) had never experienced it, while 27.1% (N=190) had a history of PROM/PPROM (Table 1).

**Table 1:** Sociodemographic characteristics, pregnancy history, and medical conditions of participants PROM, preterm rupture of membrane; PPROM, preterm premature rupture of membrane

			N/mean	%/SD
Age			36.63	9.74
Nationality		Non-Saudi	64	9.1%
		Saudi	638	90.9%
Educational level		Bachelor's	409	58.3%
		Diploma	84	12.0%
		Elementary	5	0.7%
		High school	87	12.4%
		Medium	16	2.3%
		Postgraduate studies	101	14.4%
Family income		Less than 3000	58	8.3%
		3,000-10,000	263	37.5%
		10,000-20,000	276	39.3%
		More than 20,000	105	15.0%
Job		Employee	346	49.3%
		Housewife	315	44.9%
		Student	41	5.8%
Total number of pregnancies			3.70	2.26
Number of births (≥24 weeks)			1.85	2.22
Number of miscarriages (<24 weeks)			0.74	1.30
Do you have any of these diseases?	Nothing		522	74.4%
	Urinary tract infection		86	12.3%
	Diabetes		59	8.4%
	Vaginitis or sexually transmitted infection		18	2.6%
	High blood pressure		6	0.9%
	Lupus erythematosus		2	0.3%
	Weak ovum		1	0.1%
	Uterine fibroid		1	0.1%
	Placenta previa		1	0.1%
	PCOS, obesity		1	0.1%
	Hypothyroidism		1	0.1%
	Gland and roughness		1	0.1%
	Epilepsy		1	0.1%
	Elevated liver enzymes during pregnancy		1	0.1%
	Anemia		1	0.1%
Have you ever experienced PROM/PPROM?	No		512	72.9%
	Yes		190	27.1%

Awareness, experience, and sources of information regarding chorioamnionitis

Among the participants, 375 (53.4%) had heard of CA, while 327 (46.6%) were unaware of the condition. Among those who had knowledge of CA (N=375), the most common sources of information were healthcare workers (N=110, 29.3%), followed closely by social media (N=105, 28.0%), relatives and friends who had experienced the disease (N=77, 20.5%), scientific lectures (N=45, 12.0%), and books, newspapers, and magazines (N=38, 10.1%). Regarding personal experience with CA, 110 participants (15.7%) reported having had the condition, whereas the majority (N=592, 84.3%) had not. Among those who had experienced CA (N=110), cesarean section was the predominant method of delivery (N=95, 86.4%), while natural birth was reported in 15 cases (13.6%) (Table 2).

**Table 2:** Awareness, experience, and sources of information regarding chorioamnionitis

		N	%
Have you heard of chorioamnionitis?	No	327	46.6%
	Yes	375	53.4%
If yes, what is the source? (N=375)	Healthcare workers	110	29.3%
	Social media	105	28.0%
	Relatives and friends who have been affected by this disease	77	20.5%
	Scientific lectures	45	12.0%
	Books, newspapers, and magazines	38	10.1%
Have you had chorioamnionitis?	No	592	84.3%
	Yes	110	15.7%
If yes, what was the method of delivery? (N=110)	Caesarean section	95	86.4%
	Natural birth	15	13.6%

Perceived risks and complications of chorioamnionitis for mothers and newborns

The majority of participants believed that CA increases the risk of various complications. Most respondents associated the condition with bleeding (N=623, 88.7%), emergency cesarean section (N=625, 89.0%), blood poisoning (N=530, 75.5%), high blood pressure during pregnancy (N=576, 82.1%), intrauterine fetal death (N=580, 82.6%), premature birth (N=613, 87.3%), and infection in the baby (N=519, 73.9%). Additionally, a significant proportion linked it to gestational diabetes (N=413, 58.8%), endometriosis (N=414, 59.0%), and blood clots (N=455, 64.8%). Regarding potential complications in the child, most participants believed that CA could lead to premature birth (N=630, 89.7%), sepsis (N=522, 74.4%), low birth weight (N=550, 78.3%), pneumonia (N=482, 68.7%), and death (N=530, 75.5%). Many also associated the condition with meningitis (N=435, 62.0%), retinopathy (N=421, 60.0%), and cerebral palsy (N=375, 53.4%) (Table 3).

**Table 3:** Perceived risks and complications of chorioamnionitis for mothers and newborns

	No		Yes	
	N	%	N	%
Do you think chorioamnionitis can increase the risk of these complications?				
Bleeding	79	11.3%	623	88.7%
Emergency cesarean section	77	11.0%	625	89.0%
Blood poisoning	172	24.5%	530	75.5%
High blood pressure during pregnancy	126	17.9%	576	82.1%
Intrauterine fetal death	122	17.4%	580	82.6%
Gestational diabetes	289	41.2%	413	58.8%
Premature birth	89	12.7%	613	87.3%
Endometriosis	288	41.0%	414	59.0%
Infection of the baby	183	26.1%	519	73.9%
Clots in various locations	247	35.2%	455	64.8%
Do you expect the child to have any of those complications?				
Premature birth (<37 weeks)	72	10.3%	630	89.7%
Meningitis	267	38.0%	435	62.0%
Death	172	24.5%	530	75.5%
Low birth weight	152	21.7%	550	78.3%
Retinopathy	281	40.0%	421	60.0%
Sepsis	180	25.6%	522	74.4%
Pneumonia	220	31.3%	482	68.7%
Cerebral palsy	327	46.6%	375	53.4%

Knowledge and misconceptions about chorioamnionitis among participants

The majority of participants recognized that women with a ruptured amniotic sac are at greater risk of CA (N=465, 66.2%), and many identified lower abdominal pain as a symptom (N=434, 61.8%), though a smaller proportion acknowledged an increased heart rate in the mother and fetus as a sign (N=351, 50.0%). More than half believed that inflammation of the reproductive system is a cause (N=391, 55.7%) and that the condition can lead to premature death in newborns (N=401, 57.1%), while a notable portion mistakenly thought premature babies are less likely to develop it (N=271, 38.6%). A majority agreed that CA almost always leads to preterm birth (N=397, 56.6%) and can cause serious complications (N=432, 61.5%). However, opinions were mixed on whether antibiotics alone are sufficient for treatment (N=255, 36.3%) and whether the condition can be transmitted from mother to husband (N=272, 38.7%). Regarding prevalence, only N=218 (31.1%) believed CA is common in Saudi Arabia, while N=282 (40.2%) were uncertain. Most respondents agreed that diagnosis requires specialist review (N=497, 70.8%) and that early treatment of infections can reduce incidence (N=484, 68.9%). There were misconceptions, as N=241 (34.3%) believed women cannot get pregnant again after having CA, and N=323 (46.0%) attributed the condition to poor hygiene. Additionally, N=241 (34.3%) thought it could be resolved without treatment, while N=370 (52.7%) correctly identified that the infection can be transmitted from mother to baby. There was uncertainty about whether medications for CA are more harmful than the disease itself (N=295, 42.0%) and whether it can be treated with herbs or home remedies (N=251, 35.8%). Similarly, a nearly even split existed regarding the belief that cesarean section is the only treatment (N=306, 43.6%) (Table 4).

**Table 4:** Knowledge and misconceptions about chorioamnionitis among participants

	No		I don't know		Yes	
	N	%	N	%	N	%
Women with a ruptured amniotic sac are at greater risk.	22	3.1%	215	30.6%	465	66.2%
One sign is lower abdominal pain.	45	6.4%	223	31.8%	434	61.8%
One sign is an increased heart rate (mother and fetus).	65	9.3%	286	40.7%	351	50.0%
Inflammation of the reproductive system is a cause.	69	9.8%	242	34.5%	391	55.7%
Can lead to premature death in newborns.	73	10.4%	228	32.5%	401	57.1%
Premature babies are less likely to develop it.	127	18.1%	304	43.3%	271	38.6%
Almost always leads to preterm birth.	79	11.3%	226	32.2%	397	56.6%
Antibiotics are sufficient for treatment.	143	20.4%	304	43.3%	255	36.3%
It can be transmitted from mother to husband.	144	20.5%	286	40.7%	272	38.7%
Can lead to serious complications.	52	7.4%	218	31.1%	432	61.5%
Chorioamnionitis is common in Saudi Arabia.	202	28.8%	282	40.2%	218	31.1%
Diagnosis requires a specialist review.	57	8.1%	148	21.1%	497	70.8%
Early treatment of infections reduces incidence.	55	7.8%	163	23.2%	484	68.9%
Women cannot get pregnant again after chorioamnionitis.	165	23.5%	296	42.2%	241	34.3%
Poor hygiene causes placental/amniotic sac inflammation.	120	17.1%	259	36.9%	323	46.0%
Chorioamnionitis is a simple condition that resolves without treatment.	218	31.1%	243	34.6%	241	34.3%
Infection can be transmitted from mother to baby.	78	11.1%	254	36.2%	370	52.7%
Medications for chorioamnionitis are more harmful than the disease.	97	13.8%	310	44.2%	295	42.0%
Chorioamnionitis can be treated with herbs or home remedies.	210	29.9%	241	34.3%	251	35.8%
The only treatment for chorioamnionitis is a cesarean section.	87	12.4%	309	44.0%	306	43.6%

Association between awareness of chorioamnionitis and sociodemographic characteristics

There was a significant difference in age between those who had heard of CA (median=33.00, IQR: 27.00-40.00) and those who had not (median=40.00, IQR: 31.00-45.00, p<0.001). Awareness was significantly higher among non-Saudis (N=42, 65.6%) compared to Saudis (N=333, 52.2%, p=0.048). Educational level also played a role, with higher awareness among those with postgraduate studies (N=74, 73.3%) and diploma holders (N=55, 65.5%) compared to those with a high school education (N=35, 40.2%) or lower (p<0.001). Family income was significantly associated with awareness, with lower awareness in those earning less than 3000 SAR (N=19, 32.8%) compared to those earning 10,000-20,000 SAR (N=172, 62.3%, p<0.001). Employment status showed a significant correlation, with students (N=30, 73.2%) and employees (N=208, 60.1%) being more aware than housewives (N=137, 43.5%, p<0.001). Women who had heard of CA had fewer pregnancies (median=3.00, IQR: 2.00-5.00) than those who had not (median=4.00, IQR: 2.00-6.00, p<0.001). They also had fewer births ≥24 weeks (median=0.00, IQR: 0.00-2.00) compared to those unaware (median=2.00, IQR: 0.00-4.00, p<0.001) and slightly fewer miscarriages <24 weeks (median=0.00, IQR: 0.00-1.00, p=0.040). Women who had experienced PROM/PPROM were significantly more likely to have heard of CA (N=127, 66.8%) compared to those who had not (N=248, 48.4%, p<0.001). Similarly, nearly all women who had CA (N=104, 94.5%) were aware of it, compared to only 45.8% of those who did not have the condition (N=271, p<0.001). Among those who had CA, cesarean section was significantly more common in those who were aware of the condition (N=93, 97.9%) compared to those unaware (N=2, 2.1%, p=0.003) (Table 5).

**Table 5:** Association between awareness of chorioamnionitis and sociodemographic characteristics ^U^Independent sample Mann-Whitney U test ^F^Fisher’s exact testand Fisher-Freeman-Halton exact test *p<0.05, significant PROM, preterm rupture of membrane; PPROM, preterm premature rupture of membrane

		Have you heard of chorioamnionitis?				
		No		Yes		P-value^F/U^
		N/median	Row%/IQR	N/median	Row%/IQR	
Age		40.00	31.00-45.00	33.00	27.00-40.00	<0.001*
Nationality	Non-Saudi	22	34.4%	42	65.6%	0.048*
	Saudi	305	47.8%	333	52.2%	
Educational level	Bachelor's	204	49.9%	205	50.1%	<0.001*
	Diploma	29	34.5%	55	65.5%	
	Elementary	4	80.0%	1	20.0%	
	High school	52	59.8%	35	40.2%	
	Medium	11	68.8%	5	31.3%	
	Postgraduate studies	27	26.7%	74	73.3%	
Family income	Less than 3000	39	67.2%	19	32.8%	<0.001*
	3,000-10,000	139	52.9%	124	47.1%	
	10,000-20,000	104	37.7%	172	62.3%	
	More than 20,000	45	42.9%	60	57.1%	
Job	Employee	138	39.9%	208	60.1%	<0.001*
	Housewife	178	56.5%	137	43.5%	
	Student	11	26.8%	30	73.2%	
Total number of pregnancies		4.00	2.00-6.00	3.00	2.00-5.00	<0.001*
Number of births (≥24 weeks)		2.00	0.00-4.00	0.00	0.00-2.00	<0.001*
Number of miscarriages (<24 weeks)		0.00	0.00-1.00	0.00	0.00-1.00	0.040*
Have you ever experienced PROM/PPROM?	No	264	51.6%	248	48.4%	<0.001*
	Yes	63	33.2%	127	66.8%	
Have you had chorioamnionitis?	No	321	54.2%	271	45.8%	<0.001*
	Yes	6	5.5%	104	94.5%	
If yes, what was the method of delivery? (N=110)	Caesarean section	2	2.1%	93	97.9%	0.003*
	Natural birth	4	26.7%	11	73.3%	

Association between chorioamnionitis and demographic, obstetric, and clinical factors

There was a significant difference in age between those who had experienced CA (median=34.00, IQR: 30.00-39.00) and those who had not (median=37.00, IQR: 29.00-45.00, p=0.040). Nationality was not significantly associated with CA, with similar proportions among non-Saudis (N=10, 15.6%) and Saudis (N=100, 15.7%, p=1.000). Educational level showed a significant association, with a higher prevalence among women with postgraduate studies (N=30, 29.7%) compared to those with a bachelor’s degree (N=61, 14.9%) or high school education (N=6, 6.9%, p<0.001). Family income was also significantly associated with lower rates of CA among those earning less than 3000 SAR (N=3, 5.2%) compared to those earning 10,000-20,000 SAR (N=65, 23.6%, p<0.001). Employment status played a role, with employees (N=82, 23.7%) having a significantly higher prevalence than housewives (N=26, 8.3%) and students (N=2, 4.9%, p<0.001). While the total number of pregnancies was similar between groups (p=0.545), those with CA had fewer births ≥24 weeks (median=1.00, IQR: 0.00-1.00) compared to those without the condition (median=1.00, IQR: 0.00-3.00, p<0.001). However, they had more miscarriages <24 weeks (median=1.00, IQR: 0.00-1.00) than those who did not have CA (median=0.00, IQR: 0.00-1.00, p<0.001). PROM/PPROM was significantly associated with CA, as more than half (N=101, 53.2%) of those with PROM/PPROM had experienced CA, compared to only 1.8% (N=9) of those without PROM/PPROM (p<0.001) (Table 6).

**Table 6:** Association between chorioamnionitis and demographic, obstetric, and clinical factors ^U^Independent sample Mann-Whitney U test ^F^Fisher’s exact test and Fisher-Freeman-Halton exact test *p<0.05, significant PROM, preterm rupture of membrane; PPROM, preterm premature rupture of membrane

		Have you had chorioamnionitis				
		No		Yes		P-value^F/U^
		N/median	Row%/IQR	N/median	Row%/IQR	
Age		37.00	29.00-45.00	34.00	30.00-39.00	0.040*
Nationality	Non-Saudi	54	84.4%	10	15.6%	1.000
	Saudi	538	84.3%	100	15.7%	
Educational level	Bachelor's	348	85.1%	61	14.9%	<0.001*
	Diploma	71	84.5%	13	15.5%	
	Elementary	5	100.0%	0	0.0%	
	High school	81	93.1%	6	6.9%	
	Medium	16	100.0%	0	0.0%	
	Postgraduate studies	71	70.3%	30	29.7%	
Family income	Less than 3,000	55	94.8%	3	5.2%	<0.001*
	3,000-10,000	242	92.0%	21	8.0%	
	10,000-20,000	211	76.4%	65	23.6%	
	More than 20,000	84	80.0%	21	20.0%	
Job	Employee	264	76.3%	82	23.7%	<0.001*
	Housewife	289	91.7%	26	8.3%	
	Student	39	95.1%	2	4.9%	
Total number of pregnancies		3.00	2.00-5.00	3.00	3.00-5.00	0.545
Number of births (≥24 weeks)		1.00	0.00-3.00	1.00	0.00-1.00	<0.001*
Number of miscarriages (<24 weeks)		0.00	0.00-1.00	1.00	0.00-1.00	<0.001*
Have you ever experienced PROM/PPROM?	No	503	98.2%	9	1.8%	<0.001*
	Yes	89	46.8%	101	53.2%	

Chorioamnionitis awareness and knowledge scores by demographic, health, and obstetric factors

The chorioamnionitis awareness and knowledge score varied significantly across several factors. Educational level showed a significant association (p<0.001), with those having postgraduate studies scoring higher (median=16.00, IQR: 16.00-18.00) compared to those with a high school education (median=14.00, IQR: 9.00-17.00) or medium education (median=11.50, IQR: 7.00-15.50). Family income also played a role (p=0.003), where individuals earning 10,000-20,000 SAR had a higher median score (median=16.00, IQR: 13.00-18.00) compared to those earning less than 3000 SAR (median=14.50, IQR: 10.00-17.00). A significant difference was observed in individuals who had experienced PROM/PPROM, with those who had it scoring higher (median=16.00, IQR: 15.00-18.00) compared to those who had not (median=15.00, IQR: 10.00-17.00, p<0.001). Awareness of CA was also a key factor (p<0.001), as those who had heard of it scored significantly higher (median=16.00, IQR: 15.00-18.00) than those who had not (median=12.00, IQR: 8.00-16.00). The source of information was also important (p<0.001), with individuals who learned about the condition through scientific lectures (median=16.00, IQR: 15.00-19.00) and healthcare workers (median=16.50, IQR: 16.00-18.00) scoring higher compared to those who relied on relatives or friends (median=16.00, IQR: 13.00-17.00). A significant association was found between CA history and knowledge scores (p<0.001), where those who had the condition had a higher median score (median=16.00, IQR: 16.00-18.00) compared to those who had not (median=15.00, IQR: 10.00-17.00). Additionally, among those who had CA, the method of delivery impacted the knowledge score (p<0.001), with those who underwent a cesarean section having a higher score (median=16.00, IQR: 16.00-18.00) compared to those who had a natural birth (median=16.00, IQR: 14.00-17.00) (Table 7).

**Table 7:** Chorioamnionitis awareness and knowledge scores by demographic, health, and obstetric factors ^K^Independent sample Kruskal-Wallis test ^U^Independent sample Mann-Whitney U test *p<0.05, significant PROM, preterm rupture of membrane; PPROM, preterm premature rupture of membrane

		Chorioamnionitis awareness and knowledge score (1-30)		
		Median	IQR	P-value^K/U^
Nationality	Non-Saudi	16.00	13.00-18.00	0.583
	Saudi	16.00	11.00-17.00	
Educational level	Bachelor's	16.00	11.00-17.00	<0.001*
	Diploma	16.00	12.50-18.00	
	Elementary	15.00	10.00-16.00	
	High school	14.00	9.00-17.00	
	Medium	11.50	7.00-15.50	
	Postgraduate studies	16.00	16.00-18.00	
Family income	Less than 3,000	14.50	10.00-17.00	0.003*
	3,000-10,000	15.00	10.00-18.00	
	10,000-20,000	16.00	13.00-18.00	
	More than 20,000	16.00	10.00-16.00	
Job	Employee	16.00	13.00-17.00	0.081
	Housewife	15.00	9.00-18.00	
	Student	16.00	12.00-17.00	
Do you have any of these diseases?	Nothing	16.00	10.00-17.00	0.175
	Urinary tract infection	16.00	13.00-18.00	
	Diabetes	16.00	14.00-18.00	
	Vaginitis or sexually transmitted infection	16.00	14.00-18.00	
	High blood pressure	16.00	10.00-20.00	
	Lupus erythematosus	9.50	8.00-11.00	
	Weak ovum	18.00	18.00-18.00	
	Uterine fibroid	5.00	5.00-5.00	
	Placenta previa	15.00	15.00-15.00	
	PCOS and obesity	19.00	19.00-19.00	
	Hypothyroidism	13.00	13.00-13.00	
	Gland and roughness	8.00	8.00-8.00	
	Epilepsy	15.00	15.00-15.00	
	Elevated liver enzymes during pregnancy	14.00	14.00-14.00	
	Anemia	10.00	10.00-10.00	
Have you ever experienced PROM/PPROM?	No	15.00	10.00-17.00	<0.001*
	Yes	16.00	15.00-18.00	
Have you heard of chorioamnionitis?	No	12.00	8.00-16.00	<0.001*
	Yes	16.00	15.00-18.00	
If yes, what is the source? (N=375)	Books, newspapers, and magazines	16.00	16.00-17.00	<0.001*
	Healthcare workers	16.50	16.00-18.00	
	Relatives and friends who have been affected by this disease	16.00	13.00-17.00	
	Scientific lectures	16.00	15.00-19.00	
	Social media	16.00	15.00-17.00	
Have you had chorioamnionitis?	No	15.00	10.00-17.00	<0.001*
	Yes	16.00	16.00-18.00	
If yes, what was the method of delivery? (N=110)	Caesarean section	16.00	16.00-18.00	<0.001*
	Natural birth	16.00	14.00-17.00	

Correlation between chorioamnionitis awareness and knowledge scores with age and pregnancy history

The correlation analysis between the chorioamnionitis awareness and knowledge score and various demographic and reproductive factors revealed a significant negative correlation with the number of births (≥24 weeks) (Spearman’s rho=-0.145, p<0.01), indicating that individuals with more full-term births tended to have lower awareness and knowledge scores. However, no significant correlations were observed with age (Spearman’s rho=-0.053, p=0.159), total number of pregnancies (Spearman’s rho=-0.026, p=0.494), or the number of miscarriages (<24 weeks) (Spearman’s rho=-0.065, p=0.088) (Table 8).

**Table 8:** Correlation between chorioamnionitis awareness and knowledge scores, age, and pregnancy history **Correlation is significant at the 0.01 level (2-tailed)

Correlations			
			Chorioamnionitis awareness and knowledge score (1-30)
Spearman's rho	Age	Correlation coefficient	-0.053
		Sig. (2-tailed)	0.159
		N	702
	Total number of pregnancies	Correlation coefficient	-0.026
		Sig. (2-tailed)	0.494
		N	699
	Number of births (≥24 weeks)	Correlation coefficient	-0.145**
		Sig. (2-tailed)	0.000
		N	701
	Number of miscarriages (<24 weeks)	Correlation coefficient	-0.065
		Sig. (2-tailed)	0.088
		N	699

## Discussion

This study assessed awareness of CA and its complications among women in Makkah, Saudi Arabia. The findings revealed that while more than half of the participants (53.4%) had heard of CA, a significant portion remained unaware of the condition. This lack of awareness is concerning, as CA is a serious infection that can lead to severe complications for both mothers and newborns [21]. A study conducted in Jeddah reported that 49.1% of participants had poor knowledge of CA and its complications [22]. These findings indicate a need for better public health education about infections in mothers, especially those with life-threatening conditions.

Women with postgraduate studies had the highest awareness levels (73.3%), while those with only a high school education or lower had significantly lower awareness. This trend aligns with previous studies, which have shown that individuals with higher education are more likely to seek health information and better understand medical conditions [23]. Education and health awareness are closely linked; therefore, health campaigns targeting women with less education are essential to help them obtain accurate information about pregnancy-related health risks.

Another significant finding was the role of income and employment status in awareness levels. Women with higher family income (10,000-20,000 SAR) were more aware of CA than those earning less than 3,000 SAR. Employed women and students had significantly higher awareness than housewives. These results suggest that financial stability and professional engagement may provide more opportunities for women to access healthcare information, possibly through workplace wellness programs or increased exposure to digital health resources [24]. Similar trends have been observed in previous research, where economic factors influenced maternal health knowledge and health-seeking behavior [25].

Regarding sources of information, healthcare workers (29.3%) and social media (28.0%) were the most common sources of awareness. This finding is consistent with other studies indicating that social media has become a primary channel for health information dissemination [26]. However, reliance on social media raises concerns, as misinformation can spread easily, leading to misconceptions about medical conditions. A significant proportion of participants (46%) incorrectly believed that poor hygiene was a primary cause of CA, while 34.3% thought the condition could resolve without treatment. These misconceptions highlight the importance of ensuring that health information shared on social media is accurate and evidence-based.

This study also explored the relationship between awareness and past obstetric history. Women who had experienced PROM were significantly more likely to have heard of CA than those who had not (66.8% vs. 48.4%). Nearly all women who had previously had CA (94.5%) were aware of the condition. This suggests that personal experience with pregnancy complications plays a crucial role in increasing awareness, which aligns with findings from previous research indicating that individuals are more likely to learn about medical conditions after experiencing them firsthand [27,28]. However, relying on personal experience alone is insufficient for widespread awareness, reinforcing the need for proactive health education.

The study also assessed perceptions of the risks and complications associated with CA. Most participants correctly associated CA with increased risks of emergency cesarean section (89.0%), premature birth (87.3%), and intrauterine fetal death (82.6%). However, there were misconceptions regarding treatment options, with 43.6% believing cesarean section was the only treatment and 36.3% thinking antibiotics alone were sufficient. This discrepancy suggests that while women recognize the severity of CA, they may not fully understand the clinical management of the condition. A study in the United States similarly found that although pregnant women were aware of certain pregnancy-related complications, they often lacked a comprehensive understanding of their treatment [29].

The analysis further revealed that women with a history of CA had a significantly higher rate of cesarean section delivery (97.9%) compared to those without the condition. This finding aligns with previous research indicating that CA is a major risk factor for emergency cesarean delivery due to its association with fetal distress and maternal complications [30]. The increased rate of cesarean sections among women with CA underscores the need for better preventive measures, as surgical delivery carries its own risks, including infection and prolonged recovery [31].

Women who learned about CA through healthcare workers or scientific lectures had higher knowledge scores than those who relied on social media or friends. This finding is consistent with research showing that structured educational interventions are more effective in improving health literacy than informal sources [32]. Women who had experienced PROM/PPROM or CA scored significantly higher on knowledge assessments, reinforcing the impact of direct experience in shaping health awareness.

One notable finding was the significant negative correlation between the number of full-term births and awareness scores, suggesting that women with more childbirth experiences were less knowledgeable about CA. This may stem from the perception that prior uncomplicated pregnancies indicate a lower risk of complications in subsequent pregnancies. However, this is a misconception, as multiparous women can still develop pregnancy-related infections [32]. Public health efforts should address this gap by ensuring that all pregnant women, regardless of previous pregnancy outcomes, receive adequate information about potential risks.

Strengths and limitations

This study has several strengths. It included a large sample size of 702 participants, which makes the findings more reliable. The study also covered various sociodemographic groups, allowing for a better understanding of how education, income, and employment influence awareness of CA. Additionally, the use of validated questionnaires ensured the accuracy of data collection. However, there are some limitations. Because the study used an online survey, it may have excluded women who do not use social media or have limited internet access. Another limitation is the self-reported nature of the responses, which could lead to recall bias. Furthermore, since the study was conducted in Makkah, the results may not fully represent other regions of Saudi Arabia.

Future research and recommendations

Future research should focus on developing educational programs to improve awareness of CA. Studies should explore the most effective ways to deliver health information, such as through hospitals, social media, or community workshops. Additionally, researchers should investigate how increased awareness affects pregnancy outcomes by conducting follow-up studies with pregnant women. More research is also needed to understand why misconceptions about CA persist and how they can be corrected. It would also be beneficial to conduct similar studies in other cities or rural areas to compare knowledge levels across different populations.

## Conclusions

This study found that while more than half of the women in Makkah had heard of CA, many still lacked detailed knowledge about its causes, risks, and treatment. Women with higher education, income, and previous pregnancy complications were more aware of the condition. However, misconceptions about CA were common, highlighting the need for better public health education. Healthcare professionals and social media play key roles in spreading awareness, but reliable medical information should be promoted to prevent misinformation. Increasing awareness through targeted campaigns can help reduce complications related to CA and improve maternal and newborn health outcomes.

## References

[1] Tita AT, Andrews WW (2010). Diagnosis and management of clinical chorioamnionitis. Clin Perinatol.

[2] Soper DE, Mayhall CG, Dalton HP (1989). Risk factors for intraamniotic infection: a prospective epidemiologic study. Am J Obstet Gynecol.

[3] Newton ER (1993). Chorioamnionitis and intraamniotic infection. Clin Obstet Gynecol.

[4] Newton ER, Prihoda TJ, Gibbs RS (1989). Logistic regression analysis of risk factors for intra-amniotic infection. Obstet Gynecol.

[5] Piper JM, Newton ER, Berkus MD, Peairs WA (1998). Meconium: a marker for peripartum infection. Obstet Gynecol.

[6] Tran SH, Caughey AB, Musci TJ (2003). Meconium-stained amniotic fluid is associated with puerperal infections. Am J Obstet Gynecol.

[7] Soper DE, Mayhall CG, Froggatt JW (1996). Characterization and control of intraamniotic infection in an urban teaching hospital. Am J Obstet Gynecol.

[8] Seaward PG, Hannah ME, Myhr TL (1997). International Multicentre Term Prelabor Rupture of Membranes Study: evaluation of predictors of clinical chorioamnionitis and postpartum fever in patients with prelabor rupture of membranes at term. Am J Obstet Gynecol.

[9] Rickert VI, Wiemann CM, Hankins GD, McKee JM, Berenson AB (1998). Prevalence and risk factors of chorioamnionitis among adolescents. Obstet Gynecol.

[10] Yancey MK, Duff P, Clark P, Kurtzer T, Frentzen BH, Kubilis P (1994). Peripartum infection associated with vaginal group B streptococcal colonization. Obstet Gynecol.

[11] Newton ER, Piper J, Peairs W (1997). Bacterial vaginosis and intraamniotic infection. Am J Obstet Gynecol.

[12] Abele-Horn M, Peters J, Genzel-Boroviczény O, Wolff C, Zimmermann A, Gottschling W (1997). Vaginal Ureaplasma urealyticum colonization: influence on pregnancy outcome and neonatal morbidity. Infection.

[13] Goldenberg RL, Hauth JC, Andrews WW (2000). Intrauterine infection and preterm delivery. N Engl J Med.

[14] Hillier SL, Martius J, Krohn M, Kiviat N, Holmes KK, Eschenbach DA (1988). A case-control study of chorioamnionic infection and histologic chorioamnionitis in prematurity. N Engl J Med.

[15] Aziz N, Cheng YW, Caughey AB (2009). Neonatal outcomes in the setting of preterm premature rupture of membranes complicated by chorioamnionitis. J Matern Fetal Neonatal Med.

[16] Lee SM, Park JW, Kim BJ, Park CW, Park JS, Jun JK, Yoon BH (2013). Acute histologic chorioamnionitis is a risk factor for adverse neonatal outcome in late preterm birth after preterm premature rupture of membranes. PLoS One.

[17] Metcalfe A, Lisonkova S, Sabr Y, Stritzke A, Joseph KS (2017). Neonatal respiratory morbidity following exposure to chorioamnionitis. BMC Pediatr.

[18] Venkatesh KK, Glover AV, Vladutiu CJ, Stamilio DM (2019). Association of chorioamnionitis and its duration with adverse maternal outcomes by mode of delivery: a cohort study. BJOG.

[19] Eissa GA, Bukhari AA, Alsaif BA, Abualsaud RM, Alhowaidi RM, Alshehri R (2022). Chorioamnionitis and its complications: a cross-sectional study to assess the awareness of married women in Jeddah. Cureus.

[20] (2025). General authority for the statistics of the Kingdom of Saudi Arabia population in Makkah region by gender, age group, and nationality. https://www.stats.gov.sa/en/5723.

[21] Kay VR, Liang I, Twiss J, Morais M (2024). Mode of delivery in chorioamnionitis: impact on neonatal and maternal outcomes. BMC Pregnancy Childbirth.

[22] Lamas MC, Tavares D, Mota S, Ferreira S, Amorim M (2025). Higher Education Students as Facilitators and Promoters of Health Literacy. Instructional Approaches for Health Professions Education.

[23] Hern MJ, Weitkamp T, Hillard PJ, Trigg J, Guard R (1998). Promoting women's health via the World Wide Web. J Obstet Gynecol Neonatal Nurs.

[24] Janaki S, Prabakar S (2024). Examining socioeconomic factors influencing maternal health in pregnancy. J Hum Behav Soc Environ.

[25] Jain M, Sharma PK, Kamboj K, Shyam A (2024). The impact of social media on medical education and health-care communication. J Orthop Case Rep.

[26] Kumara MG, Debelew GT, Ademe BW (2024). Lived experiences of women with uterine rupture who were managed at Nekemte specialized hospital: a qualitative study. BMC Pregnancy Childbirth.

[27] Pant A, Marschner S, Watts M, Mukherjee S, Laranjo L, Chow C, Zaman S (2023). Sociodemographic and clinical characteristics associated with improved awareness of cardiovascular disease risk in women with past pregnancy complications. Heart Lung Circ.

[28] Otu KH, Aniyete P, Annan B, Ntiamoah M, Boadum O, Antwi-Boasiako C (2023). Exploring awareness, perceptions, and barriers to seeking care for prenatal complications among pregnant women in a tertiary hospital in Ghana. Am J Clin Biomed Res.

[29] Bommarito KM, Gross GA, Willers DM, Fraser VJ, Olsen MA (2016). The effect of clinical chorioamnionitis on cesarean delivery in the United States. Health Serv Res.

[30] Güven HZ, Bornaun T (2024). Antibiotic use in cesarean procedures in developing countries: current practices and improvements. J Health Sci Med.

[31] Zace D, Orfino A, Viteritti AM, Diakanthos M, Di Pietro ML (2021). Knowledge and attitudes of young women regarding preconception health-An Italian survey. Eur J Public Health.

[32] Chen X, Gao J, Liu J (2021). Previous mode of delivery affects subsequent pregnancy outcomes: a Chinese birth register study. Ann Transl Med.

